# Strange invaders increase disturbance and promote generalists in an evolving food web

**DOI:** 10.1038/s41598-021-99843-3

**Published:** 2021-10-28

**Authors:** Jonathan R. Morris, Korinna T. Allhoff, Fernanda S. Valdovinos

**Affiliations:** 1 School for Environment and Sustainability, University of Michigan, Ann Arbor, MI USA; 2 Institute for Evolution and Ecology, Eberhard Karls University of Tübingen, Tübingen, Germany; 3 Department of Environmental Science and Policy, University of California-Davis, Davis, CA USA; 4 Department of Ecology and Evolutionary Biology, University of Michigan, Ann Arbor, MI USA; 5 Center for the Study of Complex Systems, University of Michigan, Ann Arbor, MI USA

**Keywords:** Community ecology, Ecological modelling, Ecological networks, Evolutionary ecology, Theoretical ecology

## Abstract

The patterns of diet specialization in food webs determine community structure, stability, and function. While specialists are often thought to evolve due to greater efficiency, generalists should have an advantage in systems with high levels of variability. Here we test the generalist-disturbance hypothesis using a dynamic, evolutionary food web model. Species occur along a body size axis with three traits (body size, feeding center, feeding range) that evolve independently and determine interaction strengths. Communities are assembled via ecological and evolutionary processes, where species biomass and persistence are driven by a bioenergetics model. New species are introduced either as mutants similar to parent species in the community or as invaders, with dissimilar traits. We introduced variation into communities by increasing the dissimilarity of invading species across simulations. We found that strange invaders increased the variability of communities which increased both the degree of generalism and the relative persistence of generalist species, indicating that invasion disturbance promotes the evolution of generalist species in food webs.

## Introduction

Ecologists and evolutionary biologists have long sought to understand the patterns of specialization in species across communities and the evolutionary mechanisms responsible for driving specialization^1,2^. Ecological specialization is likely a major factor in the generation and maintenance of diversity, and the subsequent stability of ecological communities^2^. This is particularly important in food webs, where species’ specialization in the form of diet breadth (i.e., number of feeding interactions) determines food web structure and, therefore, influences their stability. For example, higher levels of generalism (diet breadth) across consumers increases the connectance of food webs, which can reduce their local stability (around equilibria^3^) but increase their robustness to species extinctions^4^ and resistance to species invasions^5^. Moreover, generalism can increase species persistence when consumers are adaptive foragers^6,7^.

Numerous hypotheses have been proposed to explain the evolution of feeding specialization with many suggesting that specialists gain an adaptive advantage through greater efficiency when specialized behaviors and chemical pathways streamline the acquisition of resources^1,8,9^. For example, many insect herbivores specialize on one or a few plant families^10^, and show specialized chemical adaptations that optimize the capture, uptake, and processing of biomass acquired from the plants they consume^8^. Generalists, on the other hand, are assumed to incur trade-offs, where organisms consuming many different resources are likely to be less efficient in consuming each of them than specialized organisms with specific adaptations for their exploitation^1,11^—the classic “jack-of-all-trades, master-of-none” hypothesis^12^.

Despite the trade-offs in efficiency assumed for generalists, it is widely hypothesized that species consuming a wider range of resources will be favored over specialists when environments are more variable, making specific resources difficult to acquire^1,11^. This generalist-disturbance hypothesis suggests that communities with higher levels of environmental and community variability across time should be comprised of greater proportions of generalist species that can withstand variability by relying on other available resources when specialist species cannot. However, despite the rather intuitive nature of this hypothesis, it has proved difficult to demonstrate because of the evolutionary timescales over which these processes occur^1,11^. Some indirect evidence comes from palaeoecological records which show that specialist species, in a broad sense, are more prone to extinction under episodes of environmental change^13,14^. Modern records also indicate that specialists are disproportionately in decline in the face of global environmental change^13,15^. Small scale experimental evolution studies have demonstrated that the niche width (abiotic or biotic) of microbial organisms can be broadened within individual populations under conditions of increased variability^11^. Some theoretical studies have tested the evolutionary impact of temporal variability on simplified consumer-resource systems^16^ and in host–pathogen antagonistic systems^17^, showing that generalism within individual populations can be promoted under increased variability in abiotic resources. Despite this work, few studies have attempted to mechanistically test the impact of variability on the evolution of specialists and generalists at the scale of complex ecological communities, such as food-webs.

Here, we use an evolutionary food web model with population dynamics to introduce variation (see definition in Table 1) into communities to test the generalist-disturbance hypothesis. Our model incorporates complexity by allowing for the emergence of diverse, multi-trophic communities with continual species turnover and by including both consumer-resource and competitive interactions. While many other processes can generate variation in ecological communities, we focus on species invasion as a potential source of perturbation and variability. Invasion has long been recognized as a major driver of global change^18–20^, having effects on both ecological^21^ and evolutionary^19^ timescales. Invading species have the potential to disrupt community composition and network structure, particularly in food webs^5,21^, by exploiting enemy free niche space, serving as novel resources, and by increasing predatory and competitive pressure^21^. These effects can drive changes in the population densities of resident species, which can cascade through communities, potentially resulting in extinction^20,21^. Specifically, we tested whether invader strangeness (i.e., trait difference with respect to resident species) increases resource and community variability through time by disrupting consumer-resource dynamics and disturbing food web structure through cascading extinctions (as shown with other food web models^22^), and whether this disturbance promotes generalism (Table 1) in communities. We approached this question from a three-part hypothesis: (1) Strange invaders will increase disturbance by increasing the overall variability in resource and community biomass, and by increasing the rate of species turnover in food webs. Subsequently, this disturbance will promote the evolution of generalist consumers by increasing, (2) the degree (occurrence) and (3) relative persistence of species with larger diet breadths in communities. The three subsections of our results (see below) show findings for each of these parts of our hypothesis.

**Table 1:** Terminology and concepts.

Variation (disturbance)	In this study we consider temporal variation in basal resource biomass, community biomass, and species turnover all as indicators of disturbance. This is possible given the population and community assembly dynamics of our model; however, it does not allow for consideration of spatial variation, which may also drive patterns of feeding specialization in nature.
Specialists vs. generalists	We address feeding specialization (i.e., diet breadth) across different populations of species, where some species in our modeling framework have narrow feeding range traits (*s*) and others have larger, existing along a continuous spectrum. For our analysis we consider consumer “specialist” and “generalist” species on this continuous spectrum, but also bin species by feeding range into “specialist” and “generalist” categories to assess species lifespan and persistence differences.
Fundamental feeding range (*s*)	In our model, the fundamental feeding range of species (*s*) is fixed during the lifespan of that given species. This trait represents the width of a species feeding kernel, which accounts for fixed attack rate values on a range of potential consumed species across the body size trait axis (illustrated in Fig. 1a), regardless of what other species are present in the community at any given time. We refer to this simply as the feeding range throughout.
Realized feeding range (Proportion of community consumed)	We assess the realized feeding range of species in communities at different time outputs given their fundamental feeding range (*s*) and the composition of the rest of the community (other species consumed by the focal species). This is calculated using a threshold, where consumed species that are attacked at a rate higher than a given proportion of the focal consumer species’ maximum attack rate are counted, out of the total number of species in the community at that time. From this we calculate the proportion of community consumed by the focal consumer to estimate its realized feeding range.
Species persistence (lifespan)	We assess the persistence of generalist and specialists in food webs by tracking the lifespan of individual species across simulations. Specifically, lifespan is measured by the amount of time steps that a given species was present in a community after its initial introduction in the simulation. In this study, species lifespan and persistence are conceptually equivalent.

**Figure 1:**
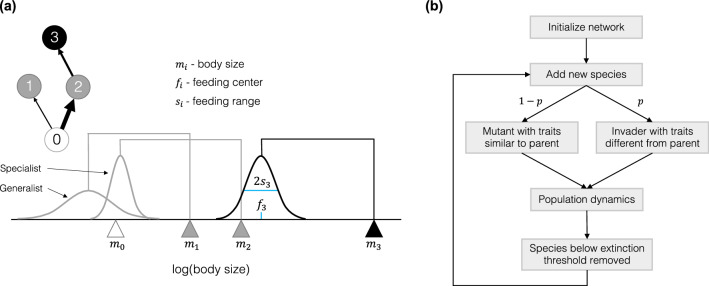
Food web network structure and community assembly process. (**a**) Species traits determine interactions and network structure. Species occupy positions on a body size (${m}_{i}$) trait axis, indicated by triangles. Species feeding kernels are illustrated as Gaussian curves, where the center of each kernel is defined by the species feeding center (${f}_{i}$) and the peak and breadth of kernels is determined by the species feeding range (${s}_{i}$). Species that fall underneath the kernels of other species are consumed by them at a rate proportional to the value of the kernel at that point on the axis. In this example, connections between species are formed given their illustrated body sizes and feeding kernels, and the resulting network structure is shown. Node 0 represents the basal resource pool in the community. Additionally, an example “specialist” and “generalist” species are illustrated, where specialist species have narrower feeding ranges, but larger potential maximum attack rates given the attack rate equation in our model (Eq. (4) in “Methods”). (**b**) Model community assembly occurs through ecological and evolutionary dynamics. Simulations are initialized with a network of two nodes, the ancestor species and the basal resource pool, and initial population dynamics are run. At fixed time intervals new species are added to the network as either mutants or invaders, determined by probability ($p$). The traits of new species are drawn probabilistically from “parent” species that already exist in the network, where mutants are more likely to have traits similar to parents and invaders have a higher probability of having traits different from parents. Between each addition of new species, the consumer-resource dynamics of the network are run, with three potential outcomes: new species go extinct as their population biomass falls below an extinction threshold, other species go extinct and are removed, or all species survive. Population dynamics and species removal occur continuously throughout the simulation, with the continued addition of new species at fixed time intervals, resulting in the overlap of ecological and evolutionary time scales in simulated communities.

Food web assembly in our model occurs through a combination of ecological and evolutionary dynamics (see “Methods”). The ecological dynamics are described by a bioenergetic consumer-resource model (Eqs. 1–6, Table 2), where species with viable biomass densities persist, while species whose biomass falls below a fixed extinction threshold are removed. Interaction strengths are governed by the feeding traits of species (body size, feeding center, feeding range), where more specialist species (narrow feeding range) have higher maximum attack rates compared to generalist species (larger feeding range) (Fig. 1a, Table 1). New species are introduced probabilistically through either mutation or invasion events (Fig. 1b). The traits of new mutant species are drawn randomly from a Gaussian distribution set around the traits of a selected extant parent species in the network. The parents of mutant species are chosen probabilistically from species with the highest population densities. Invader species traits are generated in a similar fashion but using a Gaussian distribution with a greater standard deviation, and from parent species that are selected at random with equal probability. The standard deviation of this trait range is set with the invader strangeness parameter *z*, which can be manipulated to increase the range of potential traits for invader species. Thus, a larger *z* value increases the probability that the new invader species will be “strange” compared to other species already in the community. To test the generalist-disturbance hypothesis we systematically increased this parameter for invading species across food web simulations to increase disturbance. We found that increasing the strangeness of invaders increased community and resource variability, the degree of generalism in communities, and the persistence of generalist consumers relative to specialists across time.

**Table 2:** List of model parameters and variables.

Parameter/variable	Definition
**Basal resource**	
${m}_{0}=1$	Resource body mass
${n}_{0}=1.0$	Resource input rate
$l=0.5$	Resource loss rate
**Species traits**	
${m}_{i}$	Body mass (size) ($\ge {m}_{0}$)
${f}_{i}$	Feding center
${s}_{i}$	Standard deviation of feeding range ($\ge 0.3$)
**Population dynamics**	
${B}_{0}$	Basal resource biomass density
${B}_{i}$	Consumer biomass density
${e}_{i}=0.85$	Assimilation efficiency
${g}_{ij}$	Functional response of consumer $i$ on resource $j$
${a}_{ij}$	Attack rate of consumer $i$ on resource $j$
${h}_{i}=0.4\cdot {m}_{i}^{-0.25}$	Handling time of consumer $i$
${c}_{il}$	Interspecific competition on species $i$ from species $l$
${c}_{0}=0.05$	Competition strength
${x}_{i}=0.3\cdot {{m}_{i}}^{-0.25}$	Respiration/mortality loss of consumer $i$
**Evolutionary rules**	
$p=0.2$	Probability of adding new invader species (mutant probability is $1-p$)
$z$	Invader strangeness parameter
$\varepsilon ={10}^{-8}$	Extinction threshold

If no value is provided for a parameter, it is variable.

## Results

### Do resource and community variation increase with invader strangeness?

**Figure 2:**
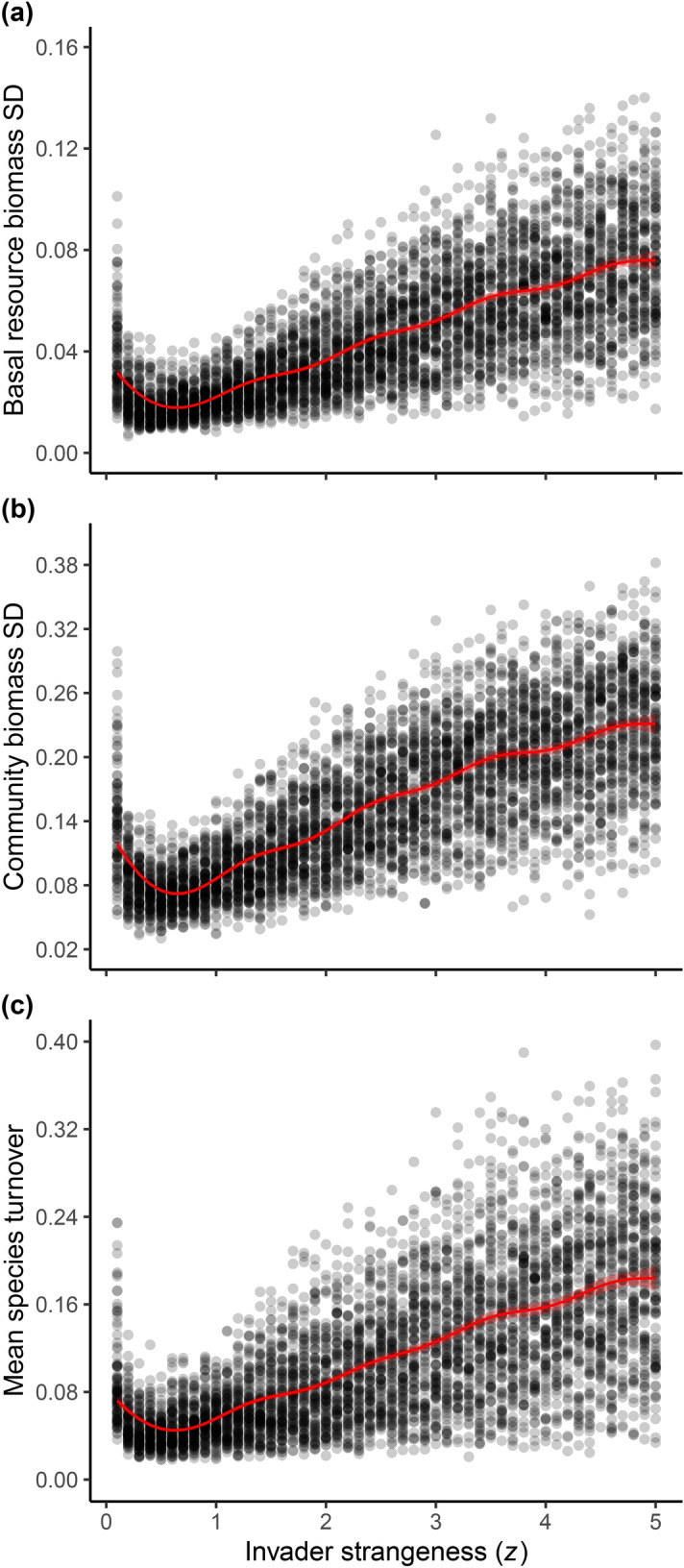
Environmental and community variation. Variation metrics show an overall increase in the variability of communities with increasing invader strangeness (*z*). (**a**) Shows the standard deviation of basal resource biomass and (**b**) community biomass across simulations. (**c**) Shows mean species turnover in communities across simulations, calculated as the proportional change in species composition in communities between time outputs (10,000 time steps). Regression lines depict the best-fit curves from generalized additive models (GAM) to account for the observed non-linearity, fit with gamma error distributions and log link functions. For all figures, 95% confidence intervals are shown by the shadded area around regression lines.

Adding increasingly strange invaders into simulated evolutionary food webs increased the variability of communities. All environmental and community variation metrics increased as invader strangeness (*z*) increased across the simulations (Fig. 2). The standard deviation (SD) of both the basal resource (Fig. 2a, Supplementary Table S1, from generalized additive model (GAM): adjusted-*R*^2^ = 0.56, generalized cross validation (GCV) = 0.12, with 61.7% deviance explained) and community biomass (Fig. 2b, Supplementary Table S1, from GAM: adjusted-*R*^2^ = 0.60, GCV = 0.08, with 62.3% deviance explained) significantly increased with the increase in invader strangeness (*z*). Moreover, mean species turnover significantly increased (Fig. 2c, Supplementary Table S1, from GAM: adjusted-*R*^2^ = 0.44, GCV = 0.20, with 49.2% deviance explained) with increasing invader strangeness (*z*), indicating strange invaders are driving more rapid extinction rates in the simulated communities.

Although the general trend of increased variability with increased invader strangeness was consistent across all metrics, across a small parameter range, for low values of *z*, the simulated communities showed more variation. This unexpected non-linearity in variation at low values of invader strangeness was found throughout our results and is likely driven by the mechanics of community assembly in our model (see “Discussion”).

### Does the degree of generalism in communities increase with invader strangeness?

**Figure 3:**
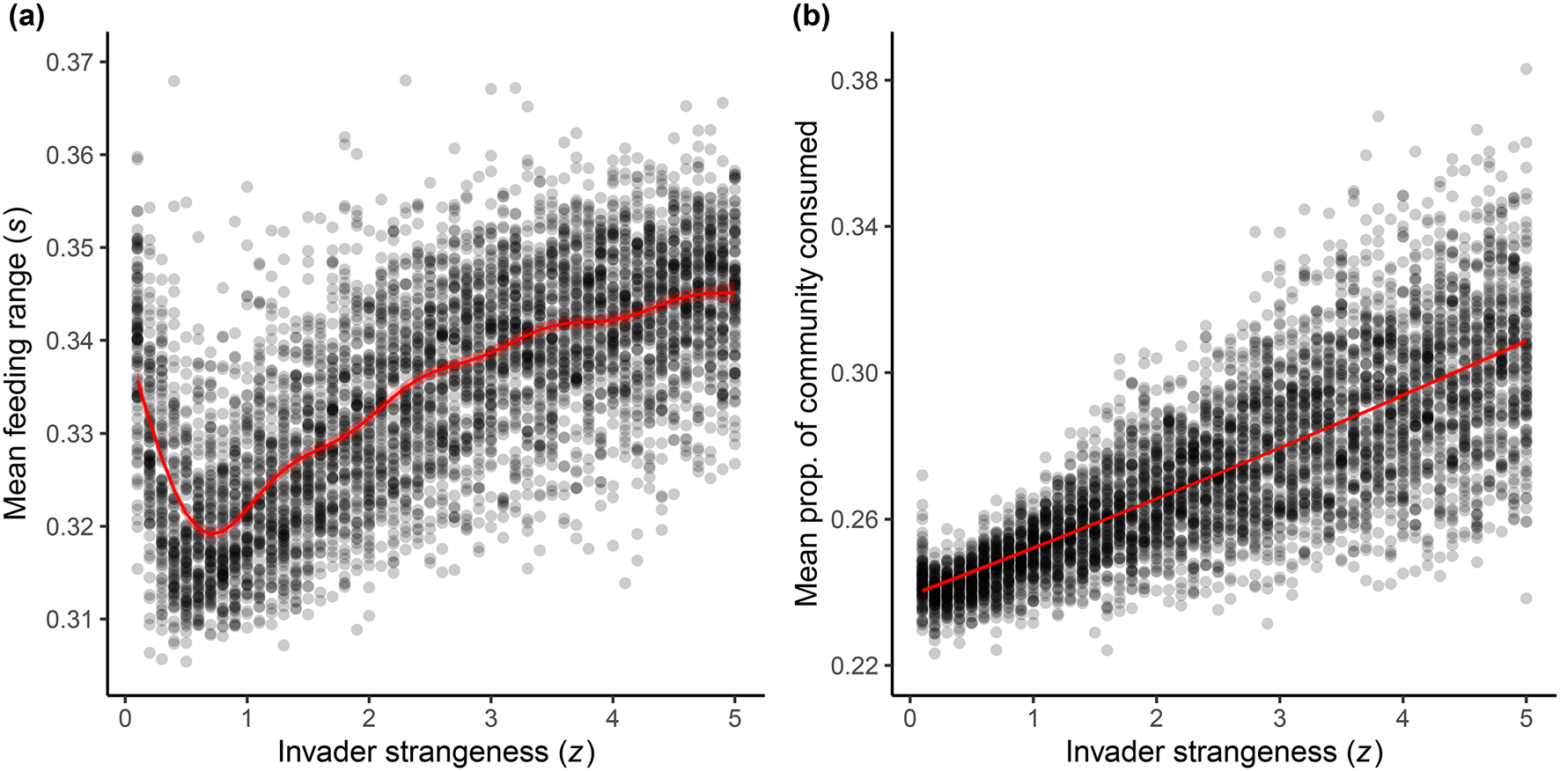
Fundamental and realized feeding range. (**a**) Shows mean fundamental feeding range (*s*) of species for all simulations across the invader strangeness (*z*) sweep. Only data from viable mutant species (mutants which did not go immediately extinct upon introduction) were assessed in order to remove the influence of invader species trait values which were directly manipulated in the experiment. The regression line depicts the best-fit curve from a generalized additive model (GAM) to account for the observed non-linear data, fit with a gamma error distribution and log link function. (**b**) Shows mean proportion of community consumed per species per simulation across the invader strangeness sweep (*z*). Data represent an estimate of the realized feeding range of all species in communities. The regression line is from a generalized linear model fit using a quasibinomial error distribution with a logit function to account for proportional data. For both figures, 95% confidence intervals are shown by the shaded area around regression lines.

Generalist occurrence showed an overall increase, measured by the mean (Fig. 3a, Supplementary Table S1, from GAM: adjusted-*R*^2^ = 0.49, GCV = 0.001, with 48.9% deviance explained) and median (Fig. S1, Supplementary Table S1, from GAM: adjusted-*R*^2^ = 0.52, GCV = 0.0001, with 52.1% deviance explained), feeding range (*s*) (Table 1) of viable mutant species in communities, indicating an increase in both the degree and proportion of species with more generalist feeding traits. The same non-linearity observed in the above variation metrics was seen in mutant feeding range data at low values of *z* (see “Discussion”).

In addition to our metrics of fundamental feeding range (*s*), the realized feeding range (Table 1) or the mean proportion of community consumed per consumer also increased with invader strangeness (Fig. 3b, Supplementary Table S1, from GLM: pseudo-*R*^2^ = 0.60). This indicates that more species were functioning as generalist consumers with greater disturbance as the proportion of connections per consumer per community increased.

### Does the persistence of generalist species increase with invader strangeness?

**Figure 4:**
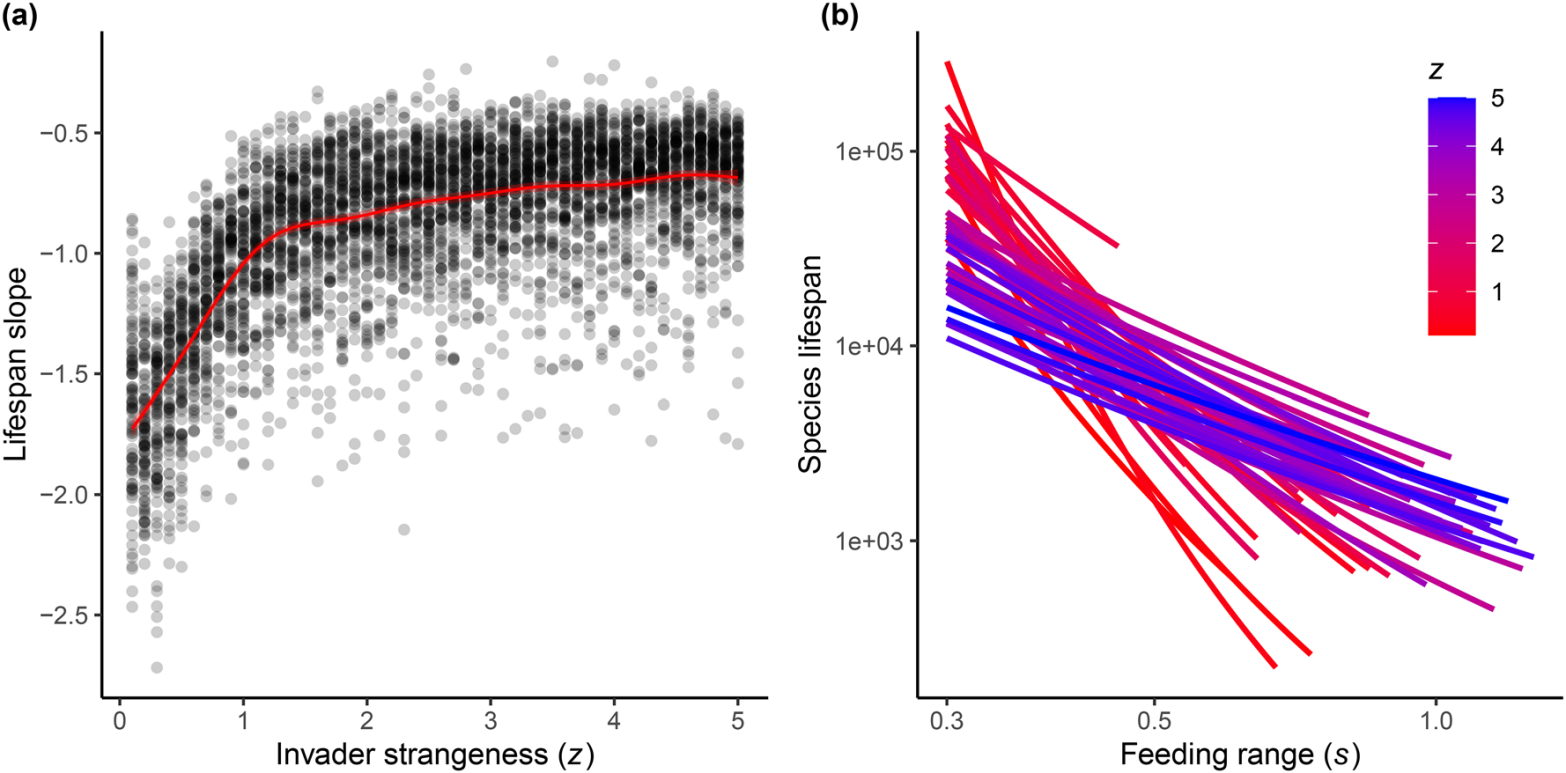
Species lifespan curves. (**a**) Shows the lifespan slope values for all simulations across the invader strangeness (*z*) sweep. Slope values are taken from the coefficients of generalized linear models (gamma error distribution and log link function) fit to the species lifespan by feeding range (*s*) data (log10-scaled) for all species in each individual simulation. The red regression line depicts the best-fit curve from our generalized additive model (GAM) to account for the observed non-linearity (identify error distribution and log link function). 95% confidence intervals shown by the shaded region around the regression line. (**b**) Shows lifespan slope curves from simulations across the invader strangeness value (*z*) for an example parameter sweep replicate. Regression lines are calculated for each individual simulation using generalized linear models with a gamma error distribution and log link function.

Both metrics of generalist persistence suggest that generalist species become more dominant in communities as invader strangeness (*z*) increases. Lifespan slopes, which represent the relationship between species lifespan and feeding range across communities for each simulation, increased, indicating that generalist species persist longer in comparison to specialist species as invader strangeness increases (Fig. 4a, Supplementary Table S1, from GAM: adjusted-*R*^2^ = 0.55, GCV = 0.06, with 55.1% deviance explained). However, this effect plateaus at higher values of *z*, indicating that the benefit to generalists of increased variation has diminishing returns when variation in the environment and the community becomes too high.

**Figure 5:**
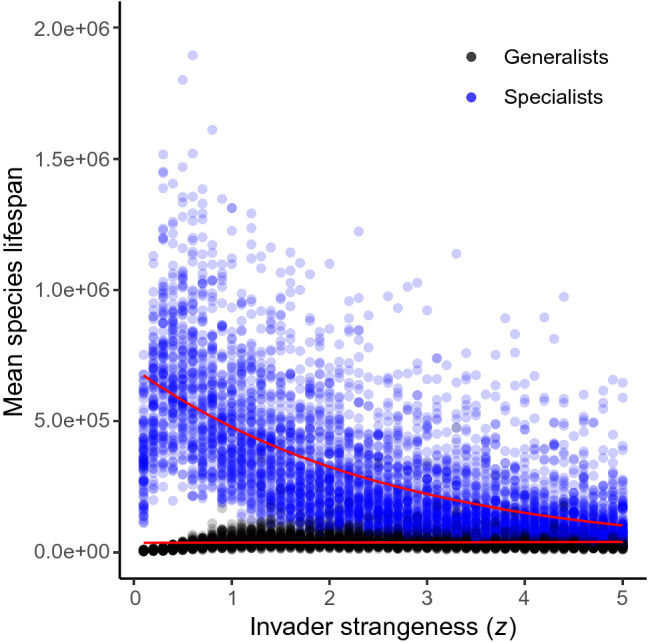
Relative persistence of generalists and specialists. Shows the mean lifespan of all species per simulation across the invader strangeness (*z*) sweep. Species are binned into “generalist” ($s\ge$ 0.39) and “specialist” ($s\le$ 0.32) categories given their feeding range (*s*) trait values. Curves (in red) are fit using a generalized linear model with a Poisson error distribution and log link function. 95% confidence intervals for the figure were too narrow to depict.

The relative persistence of generalists compared to specialists also increased with increasing invader strangeness. Specifically, the interaction between invader strangeness (*z*) and species type (generalist vs. specialist) in our model was significant, suggesting that the increase in invader strangeness affects generalists differently than specialists (Fig. 5, Supplementary Table S1, from GLM: pseudo-*R*^2^ = 0.64). Although the mean lifespan of generalists did not increase substantially across the invader strangeness parameter sweep, specialist lifespan significantly decreased, indicating that generalists persist in communities for an increasing proportion of time relative to specialists. Thus, the relative persistence of generalists compared to specialists increased with the increase in invader strangeness. Again, the observed non-linearity at low values of *z* was seen in the relative persistence data, where at very low values of *z*, specialist lifespan decreased (see “Discussion”). However, this anomaly did not impact the overall statistical trend.

## Discussion

Understanding the observed patterns of specialization across ecological communities has long been a goal of community ecology and evolutionary biology^1,2^. In consumer resource systems, it is often assumed that while specialists may be more efficient at acquiring resources, generalist species can be promoted when environmental variation is high, if they can buffer disturbance by consuming a wide range of available resources^1,8,11^. However, this hypothesis has proved difficult to demonstrate due to the complexity of studying evolutionary processes in communities over long timescales^11^. This study contributes to empirical evidence^11,13–15^ and smaller-scale theoretical work^16,17^ supporting the generalist-disturbance hypothesis by mechanistically testing the impact of variation on generalist evolution in modeled complex food webs with both species turnover and multiple interaction types. The mechanisms we evaluated include the impact of disturbance (e.g., increased variability in resource biomass) caused by invasion on populations interacting via consumer-resource and competitive interactions at a community scale, and the evolution of foraging traits (e.g., diet breadth) of resident species under those conditions through mutation and extinction.

Our results support the hypothesis that invasion increases disturbance and promotes the evolution of generalist consumers in food webs. We demonstrate that increasing the strangeness of invading species in evolving food web communities increases the overall variability of resource and community biomass (Fig. 2a,b) and the rate of species turnover (Fig. 2c). This increased level of disturbance favors the evolution of more generalist consumer species by increasing the degree of generalism in communities, assessed as both fundamental (Fig. 3a & Supplementary Fig. S1) and realized (Fig. 3b) feeding breadth, and by increasing the relative lifespan of generalists (Figs. 4 and 5). Specifically, we observed that increased disturbance selects for the persistence of species with larger feeding ranges compared to species with smaller feeding ranges, despite the model’s assumption of an efficiency-generalism trade-off^11^. Collectively, these results support the hypothesis that generalist consumer species can be selected for and maintained in complex communities with high levels of variability and disturbance.

Despite the overall increase in our variation metrics with increased invader strangeness, we observed the opposite trend at very small values of our *z* parameter (Fig. 2a–c). We suspect this non-linearity results from the community assembly processes in our model. When the invader strangeness parameter is close to $z=0.1$, the traits of invaders are drawn from a narrow range of values around parent species traits, such that “invading” species at low *z* end up being more like species that are already in the community than novel introductions. When this occurs, trophic levels (body size clusters) within communities become dominated mainly by highly efficient specialist species with a high degree of overlap in their feeding kernels (Supplementary Fig. S2). This results in a high level of resource competition, leading to occasional extinction cascades, and the larger values seen in our disturbance metrics at very low values of *z*. When invader strangeness (*z*) slightly increases from 0.1, the probability that invaders come into communities with more novel traits increases and the overlap in species feeding kernels within trophic levels is reduced, with species occupying a larger range of feeding niches (Supplementary Fig. S2). This appears to initially stabilize community dynamics and reduce variability, compared to that seen at very low values of *z*. However, as invader strangeness continues to increase beyond these low values of *z*, increasingly strange invaders begin to add disturbance to communities by occupying novel trophic positions, either as novel resources for other consumers or by occupying predator free space, leading to disrupted consumer-resource dynamics (Supplementary Fig. S2). Importantly, the non-linearity seen in our disturbance metrics is consistent throughout our data, where higher mean and median feeding range (Fig. 3a and Supplementary Fig. S1) and generalist relative persistence (Fig. 4a and 5) is also observed at these very low values of *z*—further supporting our hypothesis that increased disturbance selects for more generalized consumers.

We also observed a leveling-off in our species lifespan slope metric (Fig. 4a) at higher values of invader strangeness, indicating that after a certain level of invasion disturbance, species that have larger feeding ranges see diminishing benefits in terms of increased relative lifespan. This limit to the benefit of increased feeding generalism may be governed by the fixed influx of the basal resource in communities. Because this resource dictates the flow of energy in our communities, the total quantity available to support consumers is capped given the influx rate (${n}_{0}$) in the basal resource equation [Eq. (2) in “Methods”]. In order for generalists to benefit from the impact of variability, relative to specialists, generalist species need to have sufficient potential connections available with other resource species to buffer the effect of disturbance. If the total number of species in the community is limited by the basal resource influx, eventually, generalist consumers will be limited by the size of the community, such that increasing disturbance will no longer give generalists a relative advantage over specialists. It is also possible that the efficiency trade-off for generalist species in our model results in declining benefits as the efficiency losses from larger feeding ranges eventually outweigh the benefits gained from increased generalism (i.e., jack of all trades, master of none)^1,11,12^.

One potential limitation of our approach is the direct manipulation of the traits of invading species. Because specialist species are more efficient consumers in our model, many of the species that persist in communities tend to have small feeding ranges. As we make invaders increasingly different from what is already present in the community, introduced species may be more likely to have larger feeding range values. We managed this potential bias by assessing fundamental feeding range (*s*) values for mutant species only, which were not directly manipulated. Moreover, using species lifespan as a gauge of persistence avoids this problem by focusing on the survival of specialists and generalists regardless of their origin. That is, the survival of species in the community is determined principally by community composition and variability, and should not be biased by the traits of invading species even if more generalist species were being introduced at higher *z* values. Thus, assessing persistence of specialists and generalists based on survivorship is the most direct metric for testing our hypothesis, regardless of the source of disturbance in communities.

Our results support the long-held hypothesis of increased selection for generalists under increased environmental variation^1,11^. Further research into this question should explore other sources of variation that may be common in empirical consumer-resource communities. In addition to the disruptive potential of strange or novel invader species, the frequency of invasion itself may have significant impacts on regulating the overall level of variability in communities. This is especially pertinent given the current high rates of invasion associated with globalization^23^. Other approaches could look more generally at the impact of background environmental variability or stochasticity in communities^17^. Both seasonal and stochastic variability in energy or nutrient input in food webs can impact the birth and death rates of organisms, which will likely impact the relative persistence of generalists and specialists^17^. Ultimately, more robust conclusions can be made by comparing the results of simulation experiments like ours with empirical data that maps the feeding range traits of species in different communities^10^ and attempts to measure background levels of environmental variation, whether through invasion, seasonality, or stochasticity. Given our results, we expect that communities which are more likely to be subject to invasion from novel species (perhaps certain island and lake communities)^21^ will house a higher proportion of generalist species.

Additionally, this work further demonstrates the molding power of invasion on communities. We provide supporting evidence of invasion as a major driver of local biodiversity change by increasing variability in communities and potentially leading to increased rates of extinction^19–21^. Assuming that invader strangeness represents the functional diversity of the surrounding community, our modeling approach may also help to understand the patterns of specialization and diversity maintenance at broader spatial scales, such as metacommunities^24^. Simulation experiments with eco-evolutionary models can guide and supplement empirical evidence in understanding these questions which may be difficult to test given the long-term time scale of these processes. Our results show that invasions shape the structure of consumer-resource communities in both ecological and evolutionary time by driving species turnover and extinction rates, and ultimately, influencing the degree of feeding generalism amongst species. While the aim of this project was mainly to illuminate the impact of invasion on long-term evolutionary processes, our results also further demonstrate the importance of the active management of invasive species^20^, which may fundamentally alter the structure and stability of ecological communities.

## Methods

### Model: network structure

Communities are simulated using a modified version of the evolutionary food web models developed in Allhoff et al. (2015) and Allhoff & Drossel (2016), which build on previous models^25,26^ to show that biodiversity can be maintained in multitrophic networks despite ongoing species turnover when feeding traits are allowed to evolve independent of body mass. The model includes consumptive and competitive interactions, where interaction strengths are determined by the traits of consumer species and their resources. All species possess three traits, a body mass or size ($m$) (used interchangeably), which places them on a body size trait axis, a feeding center ($f$) and feeding range ($s$), which determine the shape and placement of their feeding curve along the axis (Fig. 1a). While the $s$ parameter specifically represents one standard deviation of a species’ feeding curve, we refer to $s$ throughout as simply the feeding range. The feeding curve represents the hypothetical, fundamental feeding niche of species and shows the potential strength of a consumer’s attack rate for a given resource located along the body size trait axis. Because interactions are determined through these Gaussian curves, our networks are technically fully connected. However, when resources are far from consumer’s feeding centers, interaction strengths become asymptotically small, having a negligible effect on dynamics. Additionally, a basal resource drives energy flow in the food web (Fig. 1a). A summary of all model parameters and variables is provided in Table 2.

### Model: population dynamics

Dynamics are governed by a bioenergetics consumer-resource model, where parameters are scaled to the body mass of species, following previous developments in Yodzis & Innes (1992) and Brose et al. (2006). The rate of change of consumer biomass (${B}_{i}$) is given by:

1 $$\frac{{dB_{i} }}{dt} = \mathop \sum \limits_{j = resources} e_{j} g_{ij} B_{i} B_{j} - \mathop \sum \limits_{j = consumers} g_{ji} B_{i} B_{j} - \mathop \sum \limits_{j = competitors} c_{ij} B_{i} B_{j} - x_{i} B_{i}$$

where ${e}_{j}$ represents the efficiency of biomass conversion of resource $j$ by consumers, ${g}_{ij}$ is the mass-specific consumption rate of resource $i$ by consumer $j$, ${c}_{ij}$ is the interference competition between consumer $i$ and $j$, and ${x}_{i}$ is the mass-specific biomass loss from respiration and mortality for consumer $i$. The rate of change in basal resource biomass (${B}_{0}$) is described by:

2 $$\frac{{dB_{0} }}{dt} = n_{0} - \mathop \sum \limits_{j = consumers} g_{j0} B_{j} B_{0} - lB_{0}$$

where ${n}_{0}$ represents the constant influx of resource biomass and $l$ the outflow rate. The time scale of the whole system is therefore defined by setting the constant resource influx rate ${n}_{0}=1$, meaning that all other rates in the system, and consequently also consumer lifespans, must be interpreted in relation to ${n}_{0}$. The basal resource is given a constant body mass trait value of ${m}_{0}=1$ which does not evolve. The mass-specific consumption rate is given by:

3 $${g}_{ij} = \frac{1}{{m}_{i}} \frac{{a}_{ij}}{1+{\sum }_{k=prey}{h}_{i}{a}_{ik}{B}_{k}}$$

where,

4 $${a}_{ij}= {m}_{i}^{0.75}\cdot {N}_{ij}={m}_{i}^{0.75}\cdot \frac{1}{{s}_{i}\sqrt{2\pi }}\cdot \mathrm{exp}\left[-\frac{{\left({log}_{10}\left({f}_{i}\right)-{log}_{10}({m}_{j})\right)}^{2}}{2{s}_{i}^{ 2}}\right]$$

describes the mass-specific attack rate of consumer $i$ on resource $j$, given the feeding kernel (${N}_{ij}$) of consumer $i$. Gaussian feeding kernels are calculated from consumer $i$’s feeding range (${s}_{i}$), feeding center (${f}_{i}$), and resource j’s body mass (${m}_{i}$), such that resources which occur close to consumer feeding center on the body size trait axis result in the highest attack rates (Fig. 1a). The mass-specific handling time for consumers is given by ${h}_{i}=0.4\cdot {m}_{i}^{-0.25}$. Interference competition between consumer $i$ and $j$ is described by:

5 $${c}_{ij}= {c}_{0}\cdot \frac{{I}_{ij}}{{I}_{ii}} \text{ for }i\ne j$$

where,

6 $${I}_{ij}= \int {N}_{ik}\cdot {N}_{jk}d\left({log}_{10}{(m}_{k})\right)$$

describes the overlap in resources $k$ between two competing consumers $i$ and $j$, such that consumers with similar feeding traits will have greater overlap between their feeding kernels resulting in higher competition coefficients.

### Model: community assembly & network evolution

Community assembly of food webs occurs through a combination of ecological and evolutionary dynamics (Fig. 1b). All ecological dynamics are described by the consumer-resource model above, where species with viable biomass densities persist in communities and species whose biomass falls below a fixed extinction threshold ($\varepsilon = {10}^{-8}$) are removed from the network. New species are introduced probabilistically into the network at fixed intervals through either mutation events ($p$) or as invaders ($1-p$), where $p$ can be manipulated to increase the frequency of either mutation or invasion events. The traits of new mutant species are drawn probabilistically from a Gaussian distribution set around the traits of a selected extant parent species in the network. Invader species traits are generated in a similar fashion but using a Gaussian distribution with a greater standard deviation. The standard deviation of this trait range is set with the invader strangeness parameter $z$, which can be manipulated to increase the range of potential traits for invader species. Thus, a larger $z$ value increases the probability that new invader species will appear “strange” compared to other species already in the community. For mutant species, $z$ is always set to 0.1.

Parents of mutants are chosen probabilistically, where species with greater individual density (species biomass/body mass) are more likely to generate new mutant species. The parents of invader species are chosen randomly, with equal probability given to all extant species in the community. Both mutants and invaders are introduced into the system at the extinction threshold biomass ($\varepsilon ={10}^{-8}$). For mutants the initial biomass is removed from the biomass of the parent species’ populations, while for invaders this biomass is added into the system without affecting the parent species’ biomass pool; however, this difference did not significantly impact our results.

Communities are initialized with a single ancestor species (starting biomass $\varepsilon ={10}^{-8}$) and the basal resource (starting biomass $=\frac{{n}_{0}}{l}=2.0$) (Fig. 1b). The ancestor species is given a body mass of $m=100$, feeding center of $f=1$, and feeding range of $s=0.4$. Upon initialization, the system is a run with only the ancestor species consuming the basal resource until a new species is introduced at 100 time steps. Thereafter, new species are introduced every 100 time steps, with ecological dynamics occurring between each species introduction. Additionally, species biomass is assessed at each 100 time step interval and non-viable species populations that fall below the biomass extinction threshold are removed. This process is repeated cyclically over the course of simulations (Fig. 1b), with many new species being generated and many removed due to extinction. The persistence of individual species is thus determined by their individual traits and overall resource availability given the composition of the rest of the community. With this dynamical approach to simulating evolving food webs, similar models have been shown to generate viable communities with both multi-trophic diversity and constant species turnover^27,28^, making this framework useful for testing the evolutionary impacts of species invasion and disturbance on community composition.

### Simulation experiments

Simulations were conducted in C, where numerical integration of differential equations was performed using the Runge–Kutta–Fehlberg algorithm from the GNU Scientific library^29^. Simulations were run for 25 million time steps, with 250,000 novel species introductions (mutants or invaders) for each simulation. To test if invasion would increase disturbance and variability in communities and drive the evolution of more generalized species, we conducted simulations where invaders were introduced with an increased probability of having trait values that were divergent from parent species. We controlled this by manipulating the invader strangeness parameter ($z$) across a range from $z=0.1$ (invader and mutant trait values are equivalent) to $z=5.0$. Invasion frequency ($p$) was fixed at 0.2 for all simulations, making mutation events more likely to occur than invasion.

We hypothesized that introducing invaders with traits that are very different from parent species and from the community should result in greater disturbance in food webs because these species would be more likely to occupy novel niche space along the body size trait axis, which could result in the overexploitation of resources either through superior feeding strategies or by allowing invaders to avoid consumption by other consumers. Together, this should increase the probability of disrupted consumer-resource dynamics and secondary species extinction occurring with the introduction of strange invaders, both resulting in increased variability of biomass in the community. As a result, this increased variation should favor the survival of more generalist species in the community if they can buffer variability by consuming a greater range of resources.

This is tested against the assumption that specialist consumers are more efficient than generalist consumers (generalist trade-off hypothesis^2,12^), which is built into our model given the formulation of the attack rate parameter ($a$), where specialist species achieve higher optimal peaks in attack rates, given their smaller feeding ranges ($s$). Thus, under conditions of low variability, our model results in communities being composed of mostly very specialized species, with narrow feeding ranges. To counter this trend toward extreme specialization, we set a floor for minimum feeding range values for all species of $s=0.3$. Given these tendencies, we expected the persistence (lifespan) of more specialist species to be greatest under conditions of low variability (low invader strangeness) and that the relative persistence of more generalist species compared to specialists should increase with disturbance due to increasingly strange invaders.

To test the robustness of these predictions, we replicated the $z$ parameter sweep 100 times using random initial seed sets, resulting in 5000 simulations total, which collectively generated over 1.25e+09 unique species across all simulations. Data from these simulations was extracted at three different time intervals. We assessed species traits and lifespan data for all species generated in simulations at every 100 time steps, excluding data from the first 50,000 time steps to avoid including transient dynamics. Community level data, including community biomass and basal resource biomass were extracted at every 50,000 time steps (excluding time 0 from analysis). Species turnover data was extracted at every 10,000 time steps. In the infrequent event that simulations did not complete (community level extinction or crashed runs) we reran simulations with different random seed sets but identical parameter values.

### Data & statistical analysis

#### Do resource and community variation increase with invader strangeness?

To assess whether the addition of increasingly strange invaders into food web communities resulted in increased variation we analyzed several metrics of community and resource variability. We calculated the standard deviation (SD) of the basal resource biomass across time for each simulation and pooled these data for all simulation replicates across the invader strangeness parameter sweep. To assess variation at the community level, we used a similar approach to calculate variability in community biomass. For this metric, we summed the population biomasses of all species in the community for each given time interval output (excluding species introduced at that time step) and calculated the SD of these values across time for each individual simulation.

Finally, to further assess community variability and to determine if increasing invader strangeness drives increased extinction in communities, we calculated species turnover for each time output. Species turnover was measured as the percentage change in the composition of species in communities between each time output (10,000 time steps). We then calculated mean species turnover over time for each simulation replicate and pooled all data together. To account for the non-linearity observed in our variation data (see “Results”) we conducted generalized additive models (GAM) to determine if increasing invader strangeness resulted in a significant increase in variability. GAMs were fit using a gamma error distribution with a log link function to account for continuous data constrained to positive values.

#### Does the degree of generalism in communities increase with invader strangeness?

To determine if the degree of generalism and the proportion of generalist species in food webs increased as invader strangeness increased, we calculated the mean and median feeding range ($s$) (Table 1) of species which occurred in communities for each simulation. We included all species that were generated and that survived for at least 100 time-steps in simulations, to remove the many non-viable species which immediately go extinct. Additionally, we included only mutant species for this metric to avoid the influence of the traits of invaders species, which we directly manipulated through the invader strangeness parameter. We reasoned this would provide a more independent metric of feeding range trends in communities. Mean and median feeding range were calculated for all simulation replicates and the impact of invader strangeness was assessed with GAMs (gamma error distribution with a log link function) to account for non-linear data (see “Results”).

Additionally, we calculated a measure of the realized feeding range of consumers (distinct from the fixed fundamental feeding range ($s$) (Table 1)) to determine if more species were functioning as feeding generalists in communities. For this metric, we calculated the attack rate of each consumer on all other species in the community (including the basal resource and the focal consumer) for each time output (every 50,000 time steps from our community data, excluding species introduced at that time step). We then calculated the proportion of the attack rate on each species compared to the focal species’ maximum possible attack rate (an ideal prey at the exact center of the consumer’s feeding kernel). We then excluded all values below a threshold of 0.1 and from this calculated the proportion of species consumed out of the total number of species in the community. This metric correlated positively with the fundamental feeding range ($s$) of consumer species (Supplementary Fig. S3) and we refer to it throughout as the realized feeding range (Table 1) of consumer species. For our statistical analysis, we calculated the mean realized feeding range of species per simulation across invader strangeness ($z$) and ran a GLM with a quasibinomial error distribution and logit link function to account for proportional data.

#### Does the persistence of generalist species increase with invader strangeness?

To determine the persistence of species in our simulations we assessed the lifespan of individual species in simulated communities across time. For a given species, lifespan was measured as the number of time steps it persisted in a simulation after its initial introduction. We used this data to determine the relationship between species persistence and feeding range traits in two ways. First, we assessed the lifespan of all species in individual simulations continuously given the feeding range trait values across species. From this, a regression coefficient was calculated from the log_10_-scaled data, using a GLM with a gamma error distribution (log link), to determine the trend or “lifespan slope” for each simulation under different levels of invader strangeness (Fig. 4b). These lifespan slope values were then assessed for all simulation replicates across the full range of the invader strangeness parameter. Because more specialized species have higher maximal attack rates and are typically more efficient in our model, we expected that the lifespans of specialist species would be longer than more generalized species and that lifespan slopes should be negative under conditions of low variation. Given this, we expected to observe a positive trend in lifespan slope values across the invader strangeness parameter sweep if disturbance was increased in simulations as $z$ became higher. We tested for this positive trend in the lifespan slope data by conducting a GAM (Gaussian distribution and the identity link function) to manage the observed non-linear trend in our data (see “Results”).

For the second approach, we aimed to determine the relative persistence of species by binning “generalist” and “specialist” species based on feeding range traits and comparing species lifespans between these groups. For this analysis we split species into bins, where specialist species included all species with feeding range $s\le$ 0.32 and generalists as all species with feeding range values $s\ge$ 0.39 (species with intermediate feeding range values were excluded from the analysis). We performed a robustness check of bin cutoffs but found no qualitative or statically significant differences in our results for a range of bin cutoff values. To assess how the relative persistence of generalists compared to specialists was influenced by invader strangeness, we then calculated the mean life span of all species falling into either of these categories per simulation and determined how these values were influenced by $z$ for all simulation replicates. To assess whether mean lifespan was different between each of these groups across the invader strangeness sweep, we conducted a GLM with species type (generalist or specialist) and invader strangeness ($z$) as fixed effect terms and tested for the statistical significance of their interaction on mean species lifespan. The GLM was run using a Poisson distribution to account for discrete lifespan count data with a log link function. All GLMs and GAMs were performed in R using the “glm” and “mgcv” functions^30^, respectively, and all non-linear parameters in GAMs were fit using generalized cross validation (GCV).

## Supplementary Information

Supplementary Information.

## Data Availability

All data used in the presented analysis are available at https://github.com/fsvaldovinos/food-web-evolution. Additional data related to this study may be requested from the authors.

## Code availability

Simulation and data analysis code for this study are publicly available at the repository https://github.com/fsvaldovinos/food-web-evolution.
