# Supplementary material for: Strange invaders increase disturbance and promote generalists in an evolving food web
Source: Sci Rep. 2021 Oct 28;11:21274. doi: 10.1038/s41598-021-99843-3 (PMC8553831; doi:10.1038/s41598-021-99843-3)
Supplement: Supplementary file 1 — Supplementary Information. [file 41598_2021_99843_MOESM1_ESM.pdf]

Strange invaders increase disturbance and promote generalists in an evolving food web

Jonathan R. Morris*, Korinna T. Allhoff, Fernanda S. Valdovinos

*Corresponding author: Dana Building, 440 Church St., Ann Arbor, MI 48109, USA
E-mail: jonno@umich.edu

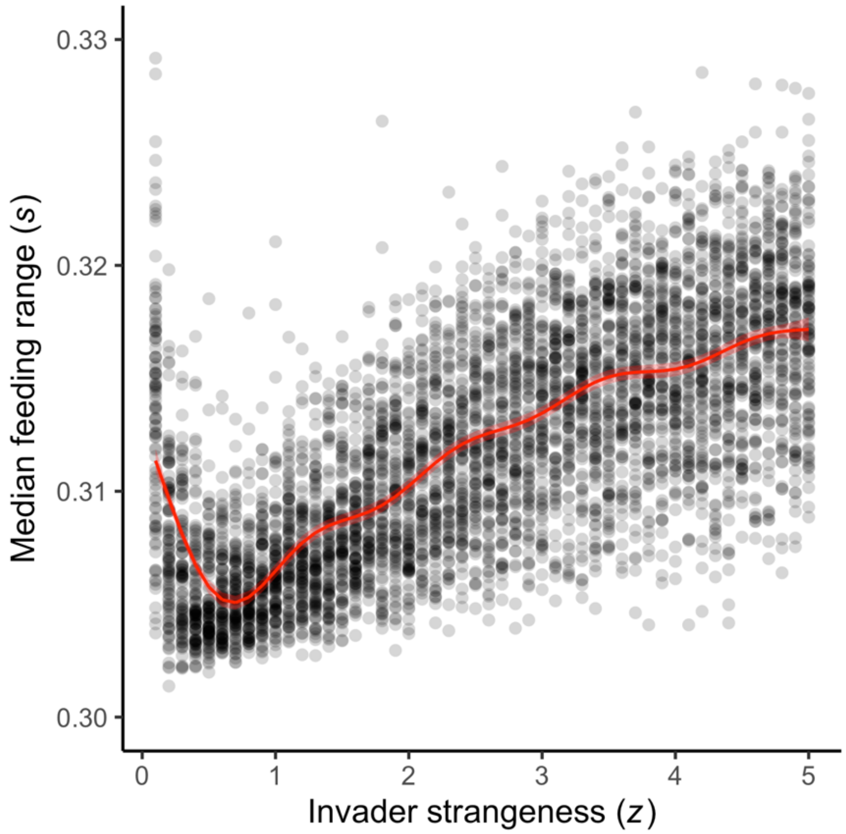

Supplementary Figure S1 – Median Fundamental Feeding Range (s). Median fundamental feeding range (s) of species for all simulations is shown across the invader strangeness (z) sweep. Only data from viable mutant species (mutants which did not go immediately extinct upon introduction) were assessed in order to remove the influence of invader species values which were directly manipulated in the experiment. The regression line depicts the best-fit curve from a generalized additive model (GAM) fit with a gamma error distribution and log link function. 95% confidence intervals are show by the shaded region around the regression line.

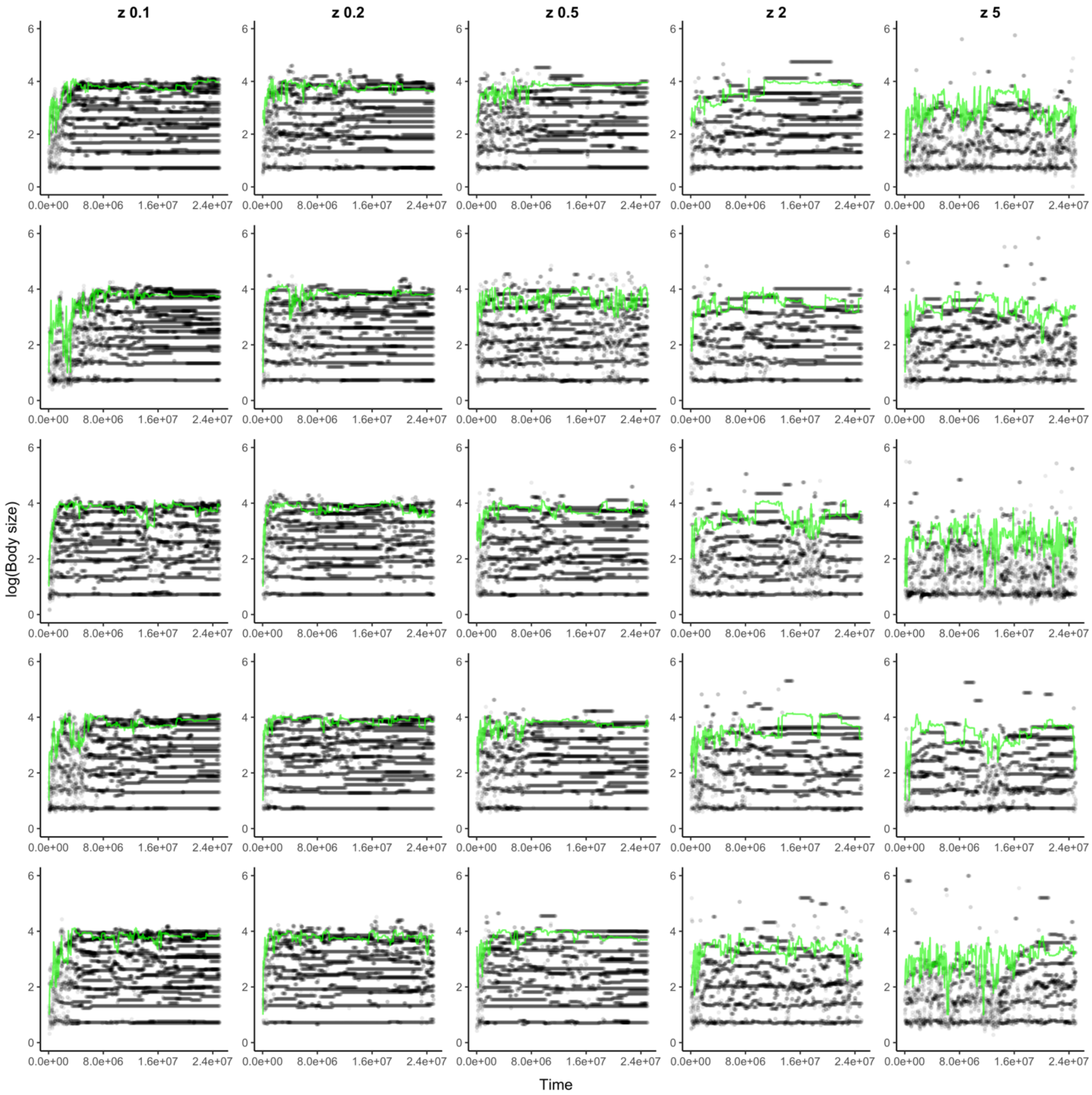

Supplementary Figure S2 - Body size across simulation time with maximum trophic position (green line). Shows species body size values for simulations from 0.1, 0.2, 0.5, 2.0, and 5.0 invader strangeness (z) values. Five example replicates for each respective value of z are shown in each column. The green line tracks the maximum trophic position (average weighted trophic position of a consumer's prey plus one) of individual species in each time output across the simulations. These figures demonstrate how communities are structured in simulations across time, with the black lines roughly representing different trophic levels in food webs. Cascading extinctions and increased disturbance can be seen when body size points vary and these lines breakdown.

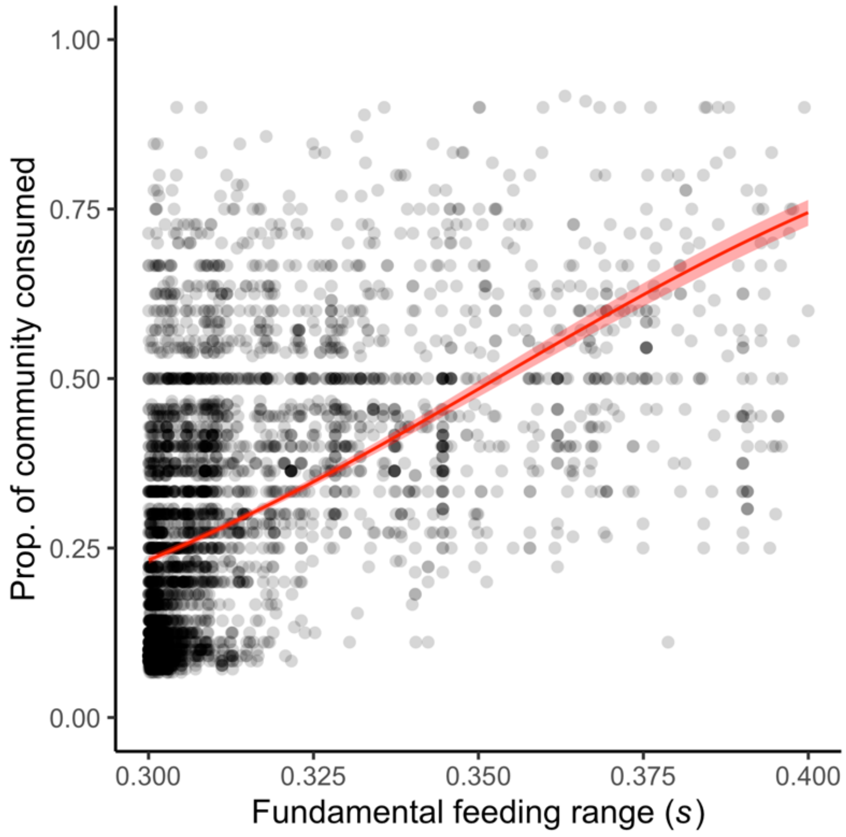

Supplementary Figure S3. Realized feeding range compared to fundamental feeding range (*s*). Data show all species extracted from community data at every 50,000 time steps and the proportion of species in the community consumed by each consumer species given their fundamental feeding range (*s*) trait value. The regression line is fit with a quasibinomial generalized linear model (GLM) with logit link function to account for proportional data. 95% confidence intervals depicted by shaded area around regression line.

Supplementary Table S1 – Results of generalized linear models (GLM) and generalized additive models (GAM) used in data analysis. Estimates and SE values not available for non-linear model terms fitted with GAMs. Asterisks represent interaction terms.

(Model Type) Parameter	Estimate	SE	t/z/F value	Pr(>|t|)
**(GAM) Resource Biomass SD~**				
Intercept	-3.170	0.00488	-650.30	<2e-16
Invader Strangeness (*z*)			995.80	<2e-16
**(GAM) Community Biomass SD~**				
Intercept	-1.926	0.00386	-498.60	<2e-16
Invader Strangeness (*z*)			1030.00	<2e-16
**(GAM) Species Turnover~**				
Intercept	-2.280	0.00606	-376.10	<2e-16
Invader Strangeness (*z*)			612.60	<2e-16
**(GAM) Mean Feeding Range (Mutants Only)~**				
Intercept	-1.095	0.00036	-3069.00	<2e-16
Invader Strangeness (*z*)			530.50	<2e-16
**(GAM) Median Feeding Range (Mutants Only)~**				
Intercept	-1.165	0.00016	-7073.00	<2e-16
Invader Strangeness (*z*)			601.90	<2e-16
**(GLM) Mean Realized Feeding Range ~**				
Intercept	-1.157	0.00239	-484.57	<2e-16
Invader Strangeness (*z*)	0.070	0.00080	87.74	<2e-16
**(GAM) Fitness Slope~**				
Intercept	-0.893	0.00351	-254.40	<2e-16
Invader Strangeness (*z*)			687.00	<2e-16
**(GLM) Relative Fitness~**				
Reference: Species Type (Generalists)				
Intercept	10.510	0.00015	70861.40	<2e-16
Invader Strangeness (*z*)	0.017	0.00005	333.80	<2e-16
Species Type (Specialists)	2.950	0.00015	19098.00	<2e-16
Invader Strangeness (*z*)*Species Type (Specialists)	-0.400	0.00005	-7452.30	<2e-16
Reference: Species Type (Specialists)				
Intercept	13.460	0.00004	311949.00	<2e-16
Invader Strangeness (*z*)	-0.384	0.00002	-19827.00	<2e-16
Species Type (Generalists)	-2.950	0.00015	-19098.00	<2e-16
Invader Strangeness (*z*)*Species Type (Generalists)	0.400	0.00005	7452.00	<2e-16
